# Ovesco StentFix for the Fixation of Fully Covered Self-Expanding Metal Stents and Lumen-Apposing Metal Stents Across Diverse Gastrointestinal Indications: A Single-Centre Pilot Case Series

**DOI:** 10.7759/cureus.111196

**Published:** 2026-06-20

**Authors:** John Ee Chew, Naomi Moy, Zaki Hamarneh, Peter Ko, Shwan Karim

**Affiliations:** 1 General Medicine, University of Queensland, Queensland, AUS; 2 Gastroenterology and Hepatology, Princess Alexandra Hospital, Brisbane, AUS

**Keywords:** endoscopic ultrasound-directed transgastric endoscopic retrograde cholangiopancreatography, fully covered self-expandable metal stent, lumen-apposing self-expanding metal stents, ovesco stentfix, stent migration, therapeutic endoscopy

## Abstract

Background and aim

Stent migration is a recognised complication of fully covered self-expandable metal stents (FCSEMS). Over-the-scope (OTS) clip-based fixation devices have emerged to address this limitation, yet real-world evidence across heterogeneous clinical indications, including lumen-apposing metal stents (LAMS) and endoscopic ultrasound-directed transgastric endoscopic retrograde cholangiopancreatography (EDGE) procedures, remains limited. We report our single-centre experience with the Ovesco StentFix system (Ovesco Endoscopy AG, Tübingen, Germany) across a broad range of gastrointestinal applications.

Methods

This retrospective single-centre case series included consecutive patients who underwent FCSEMS or LAMS fixation using the Ovesco StentFix OTS clip system at a tertiary referral centre between January 2023 and December 2025. Both benign and malignant indications were included; EDGE procedures were analysed as a distinct subgroup. The primary outcome was stent migration. Secondary outcomes included technical success, clinical success, and adverse events.

Results

Twelve patients were enrolled (median age 59 years; 58.3% male). Indications comprised benign FCSEMS applications (anastomotic leak, oesophageal perforation, post-endoscopic submucosal dissection (ESD) stricture prophylaxis, and benign stricture; n=6), malignant oesophageal obstruction (n=3), and EDGE procedures (n=3). Technical success was achieved in all patients (12/12; 100%), and clinical success was achieved in 11 of 12 patients (91.7%). Stent migration occurred in four of 12 patients (33.3%), with migration observed in two of three patients with malignant obstruction (66.7%) and two of six patients with benign indications (33.3%). No migration was observed in the EDGE subgroup. One device-related adverse event occurred in a patient whose StentFix clip became deeply embedded within the oesophageal wall and could not be retrieved at stent removal (1/12; 8.3%). Two stent-related adverse events were observed. No procedure-related mortality was recorded.

Conclusions

The Ovesco StentFix system demonstrated universal technical success across heterogeneous gastrointestinal indications, including FCSEMS for benign and malignant conditions and LAMS fixation during EDGE procedures. The overall migration rate in this small real-world cohort exceeded that of more selected published series, likely reflecting the high-risk and mixed-indication nature of the population studied. The finding of a deeply embedded clip at stent retrieval represents an important safety observation warranting further characterisation. Larger prospective studies are required to better define optimal indications and comparative effectiveness of dedicated stent fixation devices.

## Introduction

Fully covered self-expanding metal stents (FCSEMS) are widely used in the management of a variety of gastrointestinal conditions, including malignant obstruction of the gastrointestinal tract, oesophageal perforation, anastomotic leakage following upper gastrointestinal surgery (e.g., total gastrectomy or oesophagectomy), benign strictures, and fistulae [1]. FCSEMS provides a minimally invasive therapeutic option that can restore luminal patency, facilitate sealing of perforations or leaks, and allow early enteral nutrition while avoiding or delaying the need for more invasive surgical interventions [1].

Despite these advantages, a major limitation of FCSEMS is stent migration, which occurs when the stent fails to adequately embed within the gastrointestinal wall. This is particularly common with fully covered stents, as the covering prevents tissue ingrowth that would otherwise anchor the stent in place. Stent migration may lead to treatment failure, recurrent obstruction or leakage; the need for repeat endoscopic intervention; and, in some cases, distal bowel obstruction or perforation. Reported rates of FCSEMS migration vary depending on indication and anatomical location, but have been described in up to approximately 20%-40% of cases in published studies [1].

To mitigate this issue, several endoscopic techniques have been developed to anchor FCSEMS and reduce the risk of migration. These approaches include fixation using through-the-scope (TTS) clips, over-the-scope (OTS) clips, and endoscopic suturing systems, such as endoscopic full-thickness suturing devices. While these techniques have demonstrated some success, their efficacy remains variable [2-6].

More recently, a specifically designed over-the-scope fixation device, the StentFix OTS Clip System (Ovesco Endoscopy AG, Tübingen, Germany), has been introduced. This device was developed specifically to anchor gastrointestinal stents and prevent migration. Early clinical reports suggest that the StentFix system may significantly reduce stent migration and improve clinical outcomes [7-10].

However, despite increasing clinical use of this device, clear guidelines for the use of the Ovesco StentFix system have not yet been established. Current evidence remains limited and largely consists of small case series and retrospective studies [7-11]. While preliminary data suggest promising results with low migration rates and favourable technical success, the overall quality of evidence remains limited [11]. Further real-world data are needed to better define the safety, efficacy, and clinical outcomes associated with the use of the Ovesco StentFix system in routine endoscopic practice.

Therefore, this study aims to evaluate the clinical outcomes, technical success, and complication rates associated with the use of the Ovesco StentFix system for the fixation of gastrointestinal metal stents, including FCSEMS and lumen-apposing metal stents (LAMS) in gastrointestinal conditions at our institution.

## Materials and methods

Patients and methods

This retrospective single-centre case series included consecutive patients who underwent placement of an FCSEMS or LAMS with adjunctive fixation using the Ovesco StentFix OTS clip system to reduce the risk of stent migration. Procedures were performed at Princess Alexandra Hospital, a tertiary referral centre in Brisbane, Australia, between 1^st^ January 2023 and 31^st^ December 2025. 

Both benign and malignant indications were included. Indications for FCSEMS placement in this study include benign strictures, anastomotic leaks, post-endoscopic submucosal dissection (ESD) stricture prophylaxis, oesophageal perforation, and malignant obstruction. Three patients underwent endoscopic ultrasound-directed transgastric endoscopic retrograde cholangiopancreatography (EDGE), in which a LAMS was used to access the excluded stomach in the setting of Roux-en-Y gastric bypass and anchored using the StentFix system. Consecutive eligible cases during the study period were identified from a prospectively maintained endoscopy reporting system to minimise selection bias. FCSEMS (WallFlex, Agile; Boston Scientific, Marlborough, MA, USA) and LAMS (AXIOS; Boston Scientific, Marlborough, MA, USA) were deployed according to clinical indication and operator preference.

The primary outcome in this study was stent migration. Secondary outcomes were technical success, clinical success, and adverse events.

Stent migration was defined by displacement of the stent from its original position by ≥ 2cm, assessed at follow-up endoscopy, performed either routinely as scheduled or in response to patient symptoms. Time to stent migration was defined as the interval between stent fixation and the first documented evidence of stent migration on endoscopy.

Technical success was defined as the successful deployment of the StentFix device at the intended fixation site. Clinical success was defined as improvement or resolution of the underlying condition for which stent placement was performed.

For benign luminal strictures, clinical success was considered as the resolution or significant improvement of dysphagia, gastric outlet obstruction symptoms, or other obstructive symptoms, together with the ability to tolerate oral intake without the need for repeat intervention. In cases of leaks, perforations, or fistulas, clinical success was defined as complete defect closure demonstrated clinically and/or radiologically, resolution of contrast extravasation on imaging or endoscopy, and no requirement for additional endoscopic, radiological, or surgical intervention. For postoperative leaks, clinical success was characterized by the resolution of sepsis and leak-related symptoms, as well as removal of drains or cessation of drainage output attributable to the leak. In patients with malignant obstruction, clinical success was defined as improvement in obstructive symptoms and restoration of enteral nutrition or oral intake.

For EDGE procedures, clinical success was defined as successful completion of ERCP via the access tract, with or without the need for rescue access techniques (e.g., biliary rendezvous).

Follow-up duration was defined as the time from stent fixation to the last documented clinical review by the gastroenterology team or death, whichever occurred first. Patients who died after the defined follow-up period were censored at the time of their last documented gastroenterology review.

Statistical analysis was primarily descriptive. Categorical variables were summarised as frequencies and percentages, while continuous variables were reported as median (range) or mean where appropriate. Exploratory subgroup comparisons were performed descriptively, given the small sample size and limited statistical power. No formal inferential or multivariable analyses were undertaken. 

This study was approved by the Human Research Ethics Committee of Metro South Health, Brisbane, Australia (HREC Reference No. 126915). 

Procedure

All procedures were performed by experienced endoscopists using deep sedation with propofol or general anaesthesia administered by an anaesthesiologist. FCSEMS diameters and lengths were selected according to characteristics of strictures, fistulae, or perforations. All AXIOS LAMS used in the three cases were of the same diameter and size. All stent insertions were done under fluoroscopic guidance.

For FCSEMS fixation, the StentFix was first mounted onto the tip of the endoscope. The proximal edge of the stent was identified, and the clip tooth rows were aligned parallel to the stent opening to ensure secure engagement of both the stent mesh and adjacent mucosa. The clip was then deployed, and repeat fluoroscopy was performed to confirm the appropriate stent position. 

For the EDGE procedure, the excluded stomach was identified under EUS guidance and accessed using a 19-G fine needle aspiration (FNA) needle. Approximately 300 ml of saline and contrast solution was injected to achieve gastric distension, with fluoroscopic confirmation of contrast filling the excluded stomach. After confirming a suitable avascular window with Doppler and a favourable orientation toward the gastric antrum, the AXIOS LAMS was deployed to create a fistulous tract between the gastric pouch and the excluded stomach. Stent position was confirmed with EUS, fluoroscopy and drainage of the saline. The AXIOS stent was then dilated to 18mm using a controlled radial expansion (CRE) balloon under fluoroscopic guidance. A gastroscope was subsequently used to secure the stent to the gastric wall with an Ovesco StentFix clip, after which a duodenoscope was advanced through the stent to perform the endoscopic retrograde cholangiopancreatography (ERCP). 

## Results

The clinical and demographic characteristics of the 12 treated patients are shown in Table 1. The stent was positioned in the oesophagus (n=7), gastro-oesophageal junction (n=2), and gastrogastrostomy (n=3). The StentFix device was positioned at the index stent placement in all patients (n=12). 

**Table 1:** Technical success, clinical outcomes and adverse events following Ovesco StentFix fixation.

Outcome	Result	Note
Technical success	12/12 (100%)	All clips deployed successfully
Clinical success (overall)	11/12 (91.7%)	One failure: anastomotic leak (converted to endoscopic vacuum therapy)
Stent migration (overall)	4/12 (33.3%)	-
Migration in the malignant group	2/3 (66.7%)	-
Migration in the benign group	2/6 (33.3%)	One in post-endoscopic submucosal dissection and one in anastomotic leak
Mortality during follow-up	1/12 (8.3%)	Due to underlying pathology, not procedure-related
StentFix device complication	1/12 (8.3%)	Deeply embedded clip, unable to retrieve
Stent-related adverse event	2/12 (16.7%)	One in-stent stenosis (tumour ingrowth); one mild benign oesophageal stricture
Timing of stent migration, weeks (median (range))	4 (2-14)	-

Placement of the StentFix device was technically successful in all cases (100%), and clinical success was achieved in 11 of 12 patients (91.7%) (Table 2). No immediate procedure-related adverse events were observed. Stent-related adverse events occurred in two of 12 patients, while StentFix device-related adverse events were observed in one of 12 patients. Stent migration was observed in four of 12 patients (33.3%). In an exploratory analysis, stent migration was observed in two of three patients with malignancy (66.7%) and two of six patients with benign indications (33.3%). One patient died within the defined follow-up period from an underlying pathology unrelated to the procedure; no procedure-related mortality was recorded. 

**Table 2:** Baseline and clinical characteristics of patients undergoing stent fixation with Ovesco StentFix. EDGE: endoscopic ultrasound-directed transgastric endoscopic retrograde cholangiopancreatography; ESD: endoscopic submucosal dissection; FCSEMS: fully covered self-expanding metal stent; LAMS: lumen-apposing metal stent

Variable	Value (n = 12)
Age in years, median (range)	59 (27–78)
Male sex, n (%)	7 (58.3%)
Female sex, n (%)	5 (41.7%)
Indication for stenting, n (%)	
Anastomotic leak	2 (17%)
EDGE	3 (25%)
Post-ESD stricture prophylaxis	1 (8%)
Oesophageal perforation	2 (16.7%)
Benign oesophageal stricture	1 (8.3%)
Malignant oesophageal obstruction	3 (25%)
Indication category, n (%)	
Benign	6 (50%)
Malignant	3 (25%)
EDGE	3 (25%)
Stent type, n (%)	
Agile FCSEMS	7 (58.3%)
WallFlex FCSEMS	2 (16.7%)
AXIOS LAMS	3 (25%)
Follow-up duration, weeks, median (range)	7.5 (2–92)

All three EDGE procedures were performed without stent migration or device-related adverse events, with durable tract patency achieved in two cases, while one patient required endoscopic ultrasound (EUS)-guided rendezvous ERCP due to severe D2 stenosis precluding conventional cannulation, which was considered a clinical success within the predefined definition (Table 3).

**Table 3:** Outcomes of EDGE procedures with StentFix-assisted stent fixation. EDGE: endoscopic ultrasound-directed transgastric endoscopic retrograde cholangiopancreatography; AE: adverse event; ERCP: endoscopic retrograde cholangiopancreatography; EUS: endoscopic ultrasound

Patient	Indication	Migration	Stent AE	Device AE	Clinical success	Follow-up (weeks)	Notes
3	EDGE	No	None	None	Yes	9	Successful ERCP via EDGE tract
5	EDGE (necrotising pancreatitis)	No	None	None	Yes	4	Severe D2 stenosis; biliary access achieved via EUS-guided rendezvous ERCP
8	EDGE	No	None	None	Yes	42	Durable tract patency maintained

**Figure 1:**
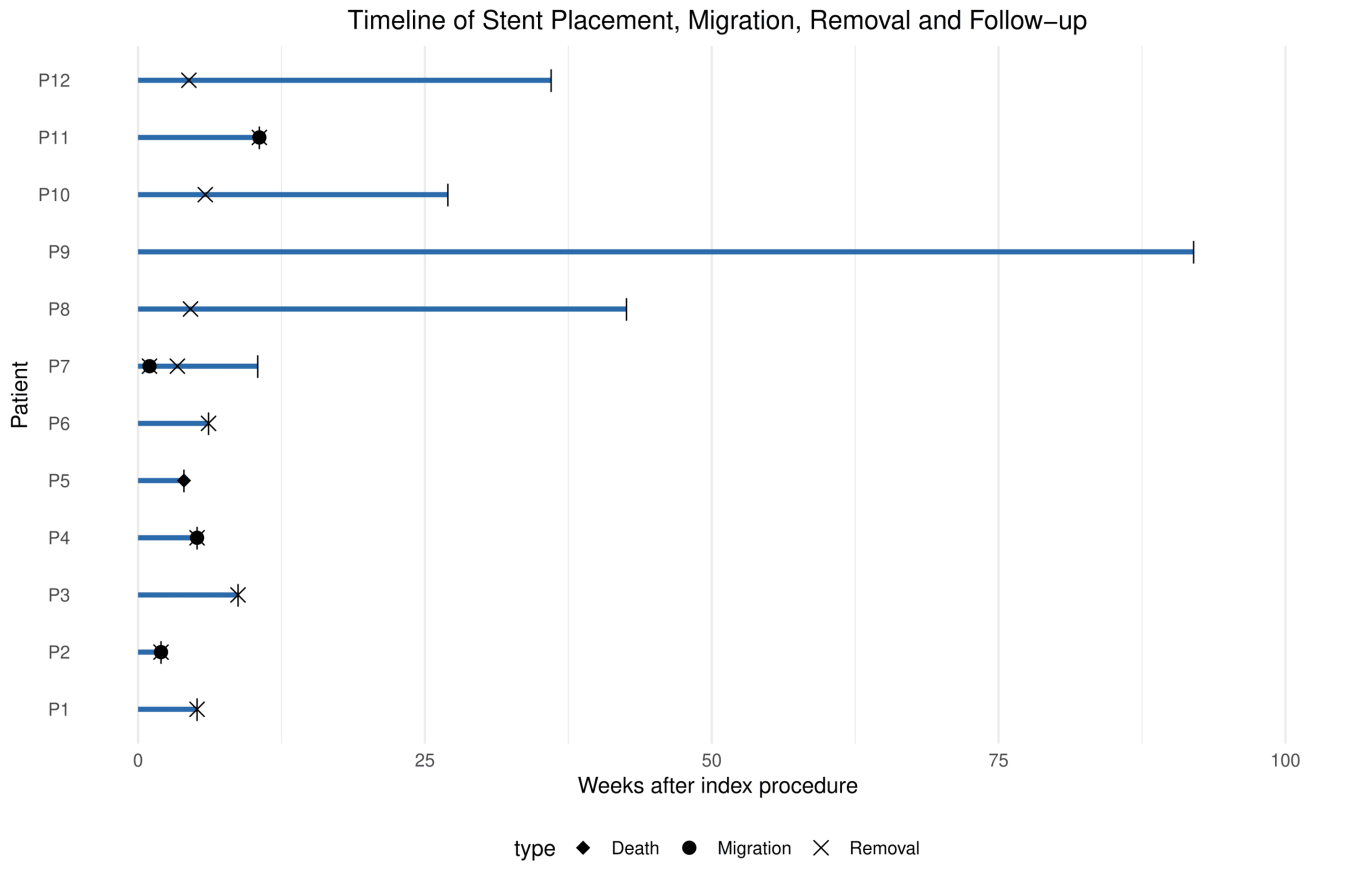
Patient-level timeline of stent fixation, migration events, adverse events and follow-up after Ovesco StentFix deployment

The timeline of Ovesco StentFix placement, stent dwell time, migration, removal, and follow-up is summarised in Figure 1.

## Discussion

Self-expandable metal stents are widely used in the management of a range of gastrointestinal conditions, including benign strictures, anastomotic leaks, perforations, and malignant obstruction. Although FCSEMS provide important therapeutic advantages due to their removability and reduced tissue ingrowth, stent migration remains a major limitation, with reported migration rates ranging from approximately 20% to 40%, particularly in benign disease where intrinsic anchoring is limited [1]. Migration may result in treatment failure, recurrent symptoms, and the need for repeat intervention, highlighting the importance of reliable fixation strategies.

Several endoscopic techniques have been developed to reduce migration risk, including TTS clips, OTS clips, and endoscopic suturing systems. While through-the-scope clips are easy to deploy, their fixation strength is limited, and their durability is variable. Endoscopic suturing provides stronger anchoring but requires specialised equipment, additional procedural time, and operator expertise. Dedicated OTS clip-based fixation devices, such as the Ovesco StentFix system, represent an important technical evolution specifically designed to engage both the stent mesh and adjacent mucosa to improve anchoring stability [2-6,11,12].

In the present single-centre case series, StentFix deployment was technically successful in all patients, with an overall clinical success rate of 91.7%. These findings are consistent with previously published studies demonstrating high technical success rates approaching 95%-100% with OTS clip-based fixation devices [8-10,13,14]. The observed migration rate of 33.3% in our cohort appears higher than that reported in earlier StentFix series; however, this likely reflects the small sample size and the heterogeneous indications included in our study population. Importantly, several patients in this cohort had recognised high-risk features for migration, including benign strictures, post-ESD defects, and stent placement across or near the gastro-oesophageal junction, a site previously identified as an independent predictor of migration [9].

Prior studies evaluating StentFix have generally reported lower migration rates. Schiemer et al. demonstrated a migration rate of approximately 8.3% in a multicentre cohort of oesophageal stents secured with a dedicated OTS clip fixation device, while Manta et al. similarly reported favourable fixation outcomes across mixed gastrointestinal indications [9,10]. However, these studies primarily involved oesophageal applications and relatively selected patient populations. In contrast, our cohort reflects real-world use across multiple anatomical sites and indications, including LAMS placed during EDGE procedures.

An additional objective of this study was to explore whether outcomes differed between benign and malignant indications. Differences in migration risk between these groups are biologically plausible, as malignant strictures may provide intrinsic anchoring through tumour ingrowth or external compression, whereas benign conditions typically lack such stabilising factors. In the present series, however, migration was observed in two of three patients with malignant obstruction (66.7%) compared with two of six benign cases (33.3%), a pattern contrary to that seen in many published series. This counterintuitive finding most likely reflects the very small number of malignant cases, as n=3 is insufficient to draw any meaningful conclusion and should be interpreted with caution.

Stent migration occurred in four patients and should be interpreted in the context of the underlying indication and procedural complexity. In one patient with malignant lower oesophageal obstruction, migration occurred early at 14 days after the patient presented with chest pain and dysphagia; the stent was subsequently replaced with a larger calibre WallFlex stent. Another migration occurred in a patient undergoing post-ESD prophylactic stenting, an indication recognised to carry a higher baseline risk of migration due to the absence of a fixed stricture. A third patient with an anastomotic leak at the gastro-oesophageal junction developed migration at approximately 24 days, necessitating transition to endoscopic vacuum therapy and representing clinical failure of stent-based therapy. Finally, late migration at approximately 10 weeks occurred in a patient with malignant mid-oesophageal obstruction. These findings highlight that migration events in this series were largely influenced by indication-specific risk factors rather than device-related failure alone [1,9-11].

One device-related complication was observed. In a patient treated for oesophageal perforation, the StentFix clip became deeply embedded within the oesophageal wall and could not be retrieved at the time of stent removal. No immediate clinical consequences were identified; however, this represents a notable safety observation that has rarely been described in existing reports of StentFix-assisted stent fixation and merits further systematic documentation across future series.

More recently, fixation of lumen-apposing metal stents using StentFix during EDGE procedures has gained increasing attention. Bronswijk et al. demonstrated that adjunctive fixation may facilitate single-session ERCP by stabilising the transgastric access tract and reducing the risk of stent dislodgement [12]. All three EDGE patients in our series underwent successful StentFix deployment with no stent migration, and clinical success was achieved in all three cases, including one patient in whom EUS-guided biliary rendezvous was required due to severe D2 stenosis. Although small, this uniformly managed EDGE subgroup demonstrates consistent anchoring performance and supports emerging evidence that dedicated fixation strategies may improve tract stability in this evolving application [12].

Several outcomes in this cohort reflected disease-related complexity rather than device failure. In a patient undergoing EDGE for necrotising pancreatitis, biliary access was ultimately achieved via a rendezvous technique despite anatomical obstruction at the second part of the duodenum. Similarly, in a patient initially treated for a presumed benign oesophageal stricture, subsequent diagnosis of oesophageal squamous cell carcinoma provided important clinical context that influenced interpretation of the treatment outcome.

The present study adds to the growing body of evidence supporting the safety and feasibility of dedicated OTS clip-based fixation devices in routine clinical practice. In particular, the inclusion of both FCSEMS and LAMS fixation across benign, malignant, and EDGE indications provides clinically relevant insight into the performance of StentFix across the heterogeneous scenarios encountered in therapeutic endoscopy.

Several limitations should be acknowledged. First, this study was retrospective and conducted at a single tertiary centre with a small sample size, limiting statistical power and generalisability. Second, there was no comparator group without fixation or using alternative fixation techniques such as suturing, precluding direct assessment of relative efficacy. Third, the heterogeneous indications and anatomical locations included in this cohort may have influenced migration outcomes. Nevertheless, the inclusion of consecutive real-world cases reflects contemporary clinical practice and provides incremental data to a still-limited evidence base. Larger prospective multicentre studies are required to better define optimal indications and comparative effectiveness of dedicated fixation devices [11, 15, 16].

## Conclusions

StentFix-assisted stent fixation was technically feasible across a heterogeneous range of gastrointestinal indications, including benign and malignant FCSEMS applications as well as lumen-apposing metal stent fixation during EDGE procedures. Technical success was achieved in all cases, supporting the practicality of dedicated over-the-scope fixation systems in routine therapeutic endoscopy. However, the migration rate observed in this real-world cohort exceeded that reported in several previously published series. This likely reflects the small sample size and the high-risk, mixed-indication nature of the study population, which included recognised migration-prone scenarios such as benign strictures, post-ESD prophylactic stenting, gastro-oesophageal junction pathology, and complex transluminal interventions. In contrast to more selected cohorts reported in the literature, the present series was intended to reflect contemporary real-world practice across diverse anatomical locations and procedural indications. Importantly, migration events appeared to be influenced more by indication-specific and anatomical risk factors than by clear device failure alone. These findings highlight the ongoing challenge of achieving durable stent anchoring in complex gastrointestinal disease and suggest that dedicated fixation devices may not uniformly overcome the intrinsic migration risk associated with certain clinical scenarios. Larger prospective multicentre studies with comparator fixation strategies are required to better define optimal patient selection, procedural indications, long-term outcomes, and the comparative effectiveness of dedicated stent fixation systems relative to alternative anchoring techniques such as endoscopic suturing or conventional OTS clip fixation.
